# Chemical control and herbicide resistance of hairy fleabane (*Erigeron bonariensis* L.) in Jordan

**DOI:** 10.1371/journal.pone.0263154

**Published:** 2023-10-12

**Authors:** Jamal R. Qasem

**Affiliations:** Plant Protection Department, Faculty of Agriculture, University of Jordan, Amman, Jordan; National Institute of Agricultural Research - INRA, MOROCCO

## Abstract

The effect of paraquat, oxadiazon and oxyfluorfen herbicides was tested on two populations of hairy fleabane (*Erigeron bonariensis* L.), collected from a date palm orchard at Tal al-Ramil (Central Jordan Valley) and al-Twal (Northern Jordan Valley) sites using the recommended rates (0.5, 1.25 and 0.792kg a.i ha^-1^ for each herbicide, respectively) and 10-fold (5, 12.50 and 7.92 kg a.i. ha^-1^, respectively) under glasshouse conditions. Results showed that the date palm weed population was resistant to the three herbicides at both application rates and al-Twal site population was highly susceptible. Two field experiments were conducted to evaluate the effectiveness of 12 herbicides in controlling the weed in the date palm orchard during the spring of 2017, revealed that *E*. *bonariensis* resists paraquat (0.5, 1.0 and 1.5 kg a.i. ha^-1^), oxadiazon (1.25 kg a.i. ha^-1^) and oxyfluorfen (0.792 kg a.i. ha^-1^) herbicides. None of the three herbicides was effective against the weed and treated plants continued to grow normally similar to those of untreated control. Ten-fold higher rates of these herbicides failed to control the weed. The effect of other tested herbicides was variable with bromoxynil plus MCPA (buctril^®^M), 2,4-D- iso-octyl ester, glyphosate, glyphosate trimesium and triclopyr being the most effective and completely controlling the weed at recommended rates of application. It is concluded that the tested populations of *E*. *bonariensis* developed resistance to paraquat, oxadiazon and oxyfluorfen but control of the weed was possible using other herbicides with different mechanisms of action. Herbicide rotation or other nonchemical weed control methods have been suggested to prevent or reduce the buildup and spread of resistant populations of this weed. These results represent the first report of herbicide resistance of *E*. *bonariensis* in Jordan.

## Introduction

Herbicides utilize synthetic and some bio-chemicals to inhibit growth or cause the death of treated plants. The impact of herbicides on the world food production is well known since their discovery in middle of the last century. However, efficacy of herbicides on weeds is much reduced with the development of herbicide-resistant weed especially when novel herbicide research and development is very limited or absent [1]. Resistance is confirmed when herbicide treated plants survive or reproduce after exposure to a rate of herbicide that is normally lethal to the wild type [1–4]. Therefore, when the same herbicide or herbicides of the same mode of action are repeatedly applied, resistant individuals increase in number and gradually dominate the site. However, the time it takes for a population shift from susceptible to totally resistant can be lengthy, depending on herbicide, environmental and plant factors [1]. The resistance problem is becoming more severe in absence of other control methods or herbicide rotation [5]. In addition, the repeated use of low rates and/or applications beyond the label recommended growth stages and the lack of tillage or other methods of control are also important contributing factors.

The genus *Erigeron* includes 50–80 species out of which almost 50 species are spreading now as weeds in more than 40 crops in 70 countries [6, 7]. These have a high fitness and can spread herbicide resistance within their population due to a combination of ecological properties such as a huge seed output, wide range of pollinating insects or self-fertilization, ability to outcross, short period for seeding, non-specific habitat requirements and long-distance seed dispersal. Of the most common and wide spread species are *Erigeron canadensis*, *E*. *bonariensis*, and *E*. *sumatrensis*. However, species of *Erigeron* include both diploids and polyploids [8] and can hybridize with each other [9].

*Erigeron bonariensis* is known to evolve resistance to glyphosate [10–16] and paraquat [17, 18] herbicides. Glyphosate resistant populations are known to require application rates 7–10 times higher than those needed to control susceptible populations in southern Spain [19]. In addition, some biotypes are known to possess herbicide resistance in different parts of the world [3, 20, 21] and variations in resistance of the weed to both herbicides have been reported [22]. *Erigeron canadensis* has been also reported to evolve resistance to glyphosate [5, 23], cloransulen-methyl, chlorimuron, imazethapyr and bispyribac [24] and paraquat [18].

In Jordan, *Erigeron* species occur in the country; are *E*. *aegyptiaca* (L.) Aiton, *E*. *albida* Sprigle, *E*. *bonariensis* L., *E*. *canadensis* (L.) Cronquist and *E*. *stricta* Willd, while *E*. *bonariensis* is the most common. It is widespread in different regions in irrigated vegetables and fruit trees, waste places and on roadsides in the Jordan Valley and high lands. The weed is an annual of 1–2 m in height, belongs to Compositae family, and produces a huge number of hairy seeds that disperse far from parent plants. It flowers from April to November under local conditions.

Recently, some of the date palm farmers in the Jordan Valley complained that *E*. *bonariensis* is irresponsive to paraquat. Paraquat was widely used in the country for general weed control and in fruit tree orchards. The chemical was banned few years ago but still available in stores of different agricultural companies and it is still marketed under other trade names. However, weed species resistance to paraquat is expected due to its wide spread use by local farmers. The herbicide is cheap and rapidly desiccates weed plants shortly after application. Therefore, low price, low application rate and rapid effects on weeds favored this herbicide by local farmers over other chemicals. This however, created serious “weed management” problems including the paraquat resistant weeds described in this study. However, farmers have tried other methods of weed control including tillage and sheep grazing but both remain ineffective because of the weed’s high seed production and its ability to re-vegetate after grazing. Cattles partially graze *E*. *bonariensis* and mostly feed on shoot tops and thus enhanced the development of auxiliary buds and branches that enable weed growth recovery and subsequent seed production.

In the last few years *C*. *bonariensis* became more abundant, invading new lands and dominating certain cultivated fields in the Jordan Valley. However, specific studies on its control or reports on its resistance to herbicides are lacking from the country. The weed has been observed occupying date palm orchards and taking over the entire fields in certain locations in the Jordan Valley. Local farmers claimed that it is not readily controlled by certain commonly used contact herbicides such as paraquat. Therefore, it was important to test the post-emergent herbicides available for use against this species. The objectives of this study were to: (1). Examine any differences in the response of selected populations of *C*. *bonariensis* in the Jordan Valley to different rates of paraquat and to other two widely used contact herbicides in the country by growing the weed in pot experiment under glasshouse conditions. (2). Further verify the actual responses of the claimed irresponsive weed population grow in one of the date palm orchards in the Jordan Valley to the tested herbicides under glasshouse conditions. (3). Testing the efficacy of other selected available foliage applied herbicides in the country of different methods of action under field conditions against this weed species.

## Materials and methods

A pot experiment was conducted under glasshouses conditions at the University of Jordan and two other experiments were performed under field conditions in the Central Jordan Valley. These were as follows:

### Glasshouse experiment

Seeds of *E*. *bonariensis* were collected from mature plants found in date palm orchards in the central Jordan Valley and al-Twal north from onion field and located in the north Jordan Valley which are almost 40 Km far apart from each other on late April 2017. Seeds of both populations were sown separately in 10 cm diameter plastic pots filled with heat-sterilized soil/peat mixture (1:1 V/V) on January 15, 2019. After emergence other weeds in all pots were hand removed. Plants of both populations were irrigated as needed under unconditioned glasshouse. At late vegetative growth stage weed plants in each population (total 40 pots each of 10 plants in average) were divided into two groups each of 20 pots that arranged in four treatments each of five pots considered as replicates. Three treatments of each population were treated each with paraquat, oxadiazon or oxyfluorfen herbicides used at the normal recommended rates (0.5, 1.25 and 0.792kg a.i ha^-1^ for each, respectively). Similar treatments were performed with the same herbicides using 5, 12.5 and 7.92 kg a.i. ha^-1^ for each, respectively. Herbicides were applied at a constant full pressure using a PVC Knapsack sprayer with a single flat type (TECS I-DH-2) nozzle and each contains 285 ml solution calculated to cover an area of 1m^2^ over which five pots of each population was spread over and treated with a single rate of one herbicide. The effect of herbicides on plants growth was visually estimated at five days after application using a scale ranged between zero to 10, at which 0 denotes that *E*. *bonariensis* plants were completely controlled and not recovered after treatment, and 10 means that no phytotoxicity effect of the herbicide was observed and plants were normally growing. Numbers between the two limits mean that tolerance/resistance to herbicides was increased with increase in visual estimation value and up to 10 indicating that weed plants were not responding to herbicide treatment, showed no phytotoxicity symptoms and kept growing unharmed similar to plants of untreated control. The same herbicides were re-applied on the same plants two weeks after the first application using the same rates used in the first spray. The effect of herbicides was visually estimated at five days after application using the same evaluation scale used before. In this experiment, each herbicide was sprayed twice and a complete coverage treatment was performed on both weed populations. Untreated pots (five pots of the fourth treatment) were included as controls and for each weed population.

### Field experiments

**Experiment 1.** The experiment was conducted on April 18, 2017 in a date palm orchard at Tal al-Ramil, in the Central Jordan Valley. The area is located at 255 m below the sea level and characterized by its tropical climate. The soil is a sandy loam of 50% sand, 25% silt and 25% clay with 1.3% organic matter content and a pH of 7.8. Date palm trees were 15 years-old and *E*. *bonariensis* was spread almost over the entire field in pure stand except in some few spots in which *Cynodon dactylon* (L.) Pers. and *Prosopis farcta* (Banks et Sol.) Macbride were found. *Erigeron bonariensis* was at a density of 30 plants m^-2^ and plants were at late vegetative or pre-flowering growth stages.

The experimental site was divided into 45 plots each of 2x2 m and 0.5 m apart, with 1 m between blocks. Twelve herbicides were used and applied in 14 treatments and an untreated control (Table 1). All herbicides are available in local markets.

**Table 1:** Herbicides tested for *Erigeron bonariensis* control in the date palm orchard in the Jordan Valley in 2017.

Common name	Trade name and a.i percentage	Chemical name	Rate of application (kg a.i. ha^-1^)	Company information
Paraquat	Gramaxon 20%	1,1’-dimethyl-(4,4’-bipyridiniom) dichloride	0.5, 1.0, 1.5	Sandoz, UK
Bromoxynil/MCPA mixture	Buctril^®^ M 20%	3,5-dibromo-4-hydroxybenzonitrile 20% (v/v) + 2-methyl-4-chlorophenoxyacetic acid (20% v/v)	0.66	May and Baker Agrochemicals (New Zealand) Ltd., Wingate, Lower Hutt, New Zealand
Ioxynil octanoate	Hocks 25%	3,5-Diiodo-4-hydroxybenzonitrile octanoate	0.825	Veterinary and Agricultural Products Mfg. Co. Ltd. Amman, Jordan
Bentazon	Basagran 48%	3-Isopropyl-2-1-3-benzo-thiadiazinon-(4)-2,2-dioxide	1.584	BASF, Germany
Oxyfluorfen	Goal 24% E	[2-chloro-N-[[4-methoxy-6-methyl-I,3,5-triazine-2-yl)-amino]carbboennyzl]esulphonamide	0.792	Rohm and Haas, Mozzata, Italy
Oxadiazon	Ronstar 25%	2-tert-butyl-4-(2,4-dichloro-5-isopropyloxyphenyl)-1,3,4-oxadiazolin-5-one	1.250	Bayer, Germany
Linuron+Ter-butryn	Tempo 13.9% EC	Linuron (13.9%) + Terbutryn 3-(3,4-dichlorophenyl)-I-methoxy-I-methyl urea plus 2-tert-butylamino-4-ethylamino-(13.9%) = (Tempo@) 6-methylthio-I,3,5-triazin	0.8062	PubChem
Glyphosate	Round up 48% (360g/L of glyphosate acid)	Isopropylamine salt of N-(phosphonomethyl) glycine	3.600	Monsanto Agriculture Company, Monsanto Europe S.A., Athens, Greece
Glyphosate trimesium	Touch down 36% (360g glyphosate/ L ammonium salt)	Trimethylsulfonium *N*-[(hydroxyphosphinato)methyl]glycine	2.204	Syngenta, Syngenta Crop Protection Pty. Ltd., Macquarie Park, NSW, Australia
2,4-D- iso-octyl ester	Esterdefore 62%	2,4-Dichlorophenoxyacetic acid isooctyl ester	2.046	Veterinary and Agricultural Products Mfg. Co. Ltd., Amman, Jordan
Mecoprop	CMPP 60%	a-(2-Methyl-4-chlor-phenoxy)-propionic acid	1.980	Atlas Interlates Ltd.
Triclopyr	Garlon 4E 61.6%	2-[(3,5,6-trichloro-2-pyridinyl)oxy]acetic acid	2.563	Dow Chemical Company LLC, Indianapolis, IN,120 USA

The herbicides were applied as directed spray on the foliage parts of plants at late vegetative to pre-flowering stage and at a constant full pressure in the morning in absence of air currents and thus any possible chemical drift, using a Knapsack sprayer with a single nozzle at a volume rate of 1083.3 l ha^_1^. Visual estimation of the effects of these chemicals on weed growth was carried out on May 3^rd^ and 11^th^, 2017 using the same scale used for evaluation of the herbicides effects in the glasshouse experiment. The evaluation was carried out by three persons and the average of the three scores was considered for each plot.

**Experiment 2.** The same procedure was followed as above in experiment 1, but only paraquat, oxyfluorfen and oxadiazon herbicides were re-tested since the weed was not affected by these chemicals in the first experiment. The three herbicides were applied at ten times higher rates (5, 12.5 and 7.92 kg a.i ha^-1^, respectively) on 18 May 2017 and similar scale of evaluation and estimation of the herbicides effects on the weed were carried out two weeks after application. Treatments replications and the experimental design were the same as for experiment 1. However, weed plants were between late vegetative to flowering growth stages.

* The reported work received no funds from any source or foundation and thus no permission from any is required prior to its publication.

### Statistics

Glasshouse and field experiments were laid out in a randomized complete block design with 5 replications for treatments in glasshouse experiment and 3 replications for each treatment in field experiments. Visual estimation scores on herbicides effects on weed populations and in all experiments were subjected to normality test first then to the analysis of variance (ANOVA) using SAS software SAS (r) version 9.1 [25]. Treatments means were separated and compared using the least significant difference test (LSD *p* = 0.05).

## Results

### Glasshouse experiment

The effects of paraquat, oxadiazon and oxyfluorfen on *E*. *bonariensis* used at normal and high rates of application were shown in Table 2.

**Table 2:** The effects of low and high rates of application of three herbicides on two populations of *Erigeron bonariensis* in pot experiment and sprayed twice at 15 day interval at late vegetative growth stage.

Treatments	Normal rates of application (a.i kgha^-1^)	First spray	Second spray	High rates of application (a.i kgha^-1^)	First spray	Second spray
		Score out of control	Score out of control		Score out of control	Score out of control
Date Palm Resistant Population of *E*. *bonariensis*						
Control	0.000	10.0^a^	10.0^a^	0.00	10.0^a^	10.0^a^
Paraquat	0.500	9.2^a^	7.8^b^	5.00	6.4^d^	4.6^c^
Oxyfluorfen	0.792	9.2^a^	8.4^ab^	7.92	8.0^bc^	7.4^b^
Oxadiazon	1.250	9.4^a^	8.4^ab^	12.50	8.4^ab^	7.2^b^
Al-Twal Susceptible Population of *E*. *bonariensis*						
Control	0.000	10.0^a^	10.0^a^	0.00	10.0^a^	10.0^a^
Paraquat	0.500	0.8^c^	0.0^d^	5.00	1.2^f^	1.0^d^
Oxyfluorfen	0.792	0.6^c^	0.0^d^	7.92	3.4^e^	1.0^d^
Oxadiazon	1.250	8.2^b^	2.4^c^	12.50	7.2^cd^	2.4^d^
LSD (p≤0.05)	-	0.96	1.7	-	1.9	1.4

Mean values for five replicate pots treated with each herbicide on a scale of 0–10 where 0 denotes that the weed was completely controlled and the weed had no possible recovery after treatment, and 10 means that the weed was in full growth and showed no herbicide phytotoxicity. Mean values in the same column for both weed populations followed by the same lower-case letter are not significantly different according to Fisher’s LSD at *P* = 0.05.

**Fig 1:**
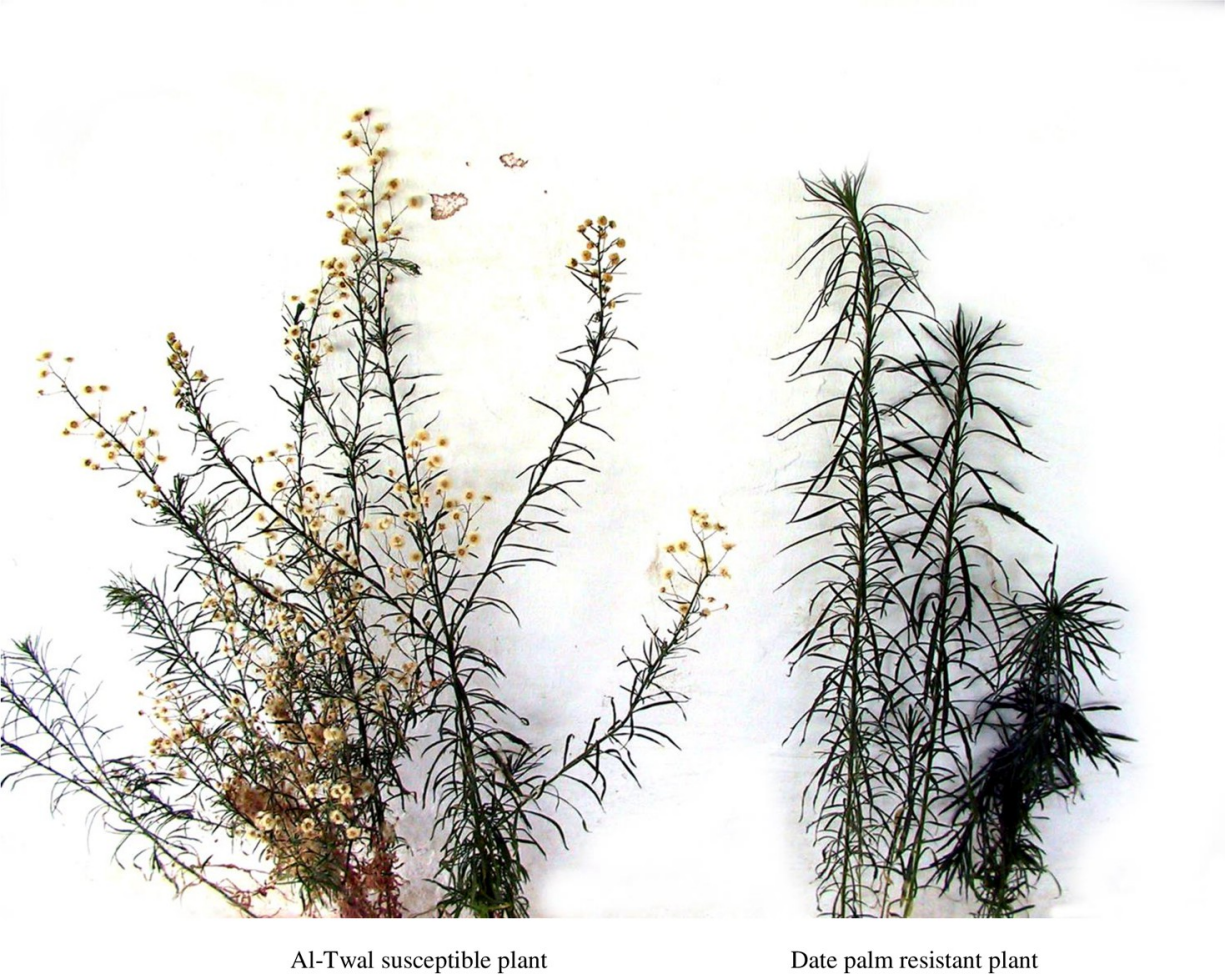
*Erigeron bonariensis*, herbicide–susceptible plant raised from seeds of al-Twal site population (left) and herbicide-resistant plant raised from seeds collected of the date palm orchard population (right) located in the Jordan valley.

*Erigeron bonariensis* was not affected by any of the herbicides at normal rates in the first application (9.2, 9.2 and 9.4 for paraquat, oxyfluorfen and oxadiazon, respectively) and at second application (7.8, 8.4 and 8.4, respectively) (Table 2). Treated plants grew more or less similar to those of untreated control although phytotoxicity effects of the three herbicides in the second spray were slightly higher. At a 10-folds application rates more suppressive effect on weed was observed (6.4, 8.0 and 8.4 for the three herbicides, respectively) at first spray and 4.6, 7.4 and 7.2 at the second application, respectively. However, none of the herbicides effectively controlled the weed at any rate used. Repeated treatments of the same herbicides on weed plants of date palm population resulted in some more phytotoxic effects especially with paraquat but plants almost recovered normally thereafter (Table 2). In contrast, at normal recommended rates of application, weed plants raised from al-Twal population seeds were almost completely controlled at first application with paraquat (0.8) and oxyfluorfen (0.6) but not so with oxadiazon (8.2). At second application, the weed was completely controlled with paraquat (0) and oxyfluorfen (0) and severely reduced with oxadiazon (2.4). At 10-folds higher rates in first spray scores were 1.2, 3.4 and 7.2, for paraquat, oxyfluorfen and oxadiazon, respectively. In the second application, the herbicides scored 1.0, 1,0 and 2.4. Herbicide-resistant plants were not branching, but late flowering with low number of flowers that produced low number of seeds compared with herbicides-susceptible plants (Fig 1).

### Field experiments

**Experiment 1.** The effects of tested herbicides on *E*. *bonariensis* were visually estimated in the field at 2 and 3 weeks after treatments. Herbicides were greatly varied in their effects on the weed (Table 3).

**Table 3:** Visual estimations of the effects of foliage applied herbicides on *Erigeron bonariensis* control, values are average scores of two estimations at 15 and 23 days after herbicides application (DAT).

Herbicide	Score at 15 DAT	Score at 23 DAT
Untreated (Control)	10.0^a^	10.0^a^
Paraquat (0.5kg a.i.ha^-1^)	10.0^a^	10.0^a^
Paraquat (1kg a.i. ha^-1^)	10.0^a^	10.0^a^
Paraquat (1.5 kg a.i.ha^-1^)	9.0^ab^	10.0^a^
Bromoxynil/MCPA mixture	0.0^e^	0.0^e^
Ioxynil octanoate	3.0^cd^	1.0^d^
Bentazon	3.8^c^	3.0^c^
Oxyfluorfen	9.8^a^	10.0^a^
Oxadiazon	7.8^b^	9.5^a^
Linuron+terbutryn	7.8^b^	5.5^b^
Glyphosate	1.8^de^	0.0^e^
Glyphosate trimesium	1.8^de^	1.0^d^
2,4-D- iso-octyl ester	0.8^e^	0.3^de^
Mecoprop	7.3^b^	2.8^c^
Triclopyr	1.0^e^	0.0^e^
LSD ≤ *p* (0.05)	1.8	1.0

Mean values for three replicates treated with each herbicide using a scale of 0–10 where 0 denotes that the weed was completely controlled and had no possible recovery after treatment, and 10 means that the weed was in full growth and no herbicide phytotoxicity observed per plot. Mean values in the same column followed by the same lower-case letter are not significantly different according to Fisher’s LSD at *P* = 0.05.

Bromoxynil/MCPA mixture (Buctril^®^M) was first (0) in controlling the weed and totally controlled all plants in treated plots 15 days after application. Other herbicides were highly effective and their phyotoxicity visually estimated values were not significantly different from that of bromoxynil/MCPA mixture including 2,4-D- iso-octyl ester (0.8), triclopyr (1.0), glyphosate (isopropylamine salt of N-(phosphonomethyl) glycine) scored and glyphosate trimesium (Trimethylsulfonium *N*-[(hydroxyphosphinato) methyl] glycine) (1.8 for each). The ioxynil octanoate and bentazon showed moderate effects (3 and 3.8, respectively) while paraquat, oxadiazon and oxyfluorfen failed to affect the weed (10, 7.8 and 9.8, respectively) and to a less extent were linuron + terbutryn (Tempo*) (7.8) and mecoprop (7.3) herbicides.

**Fig 2:**
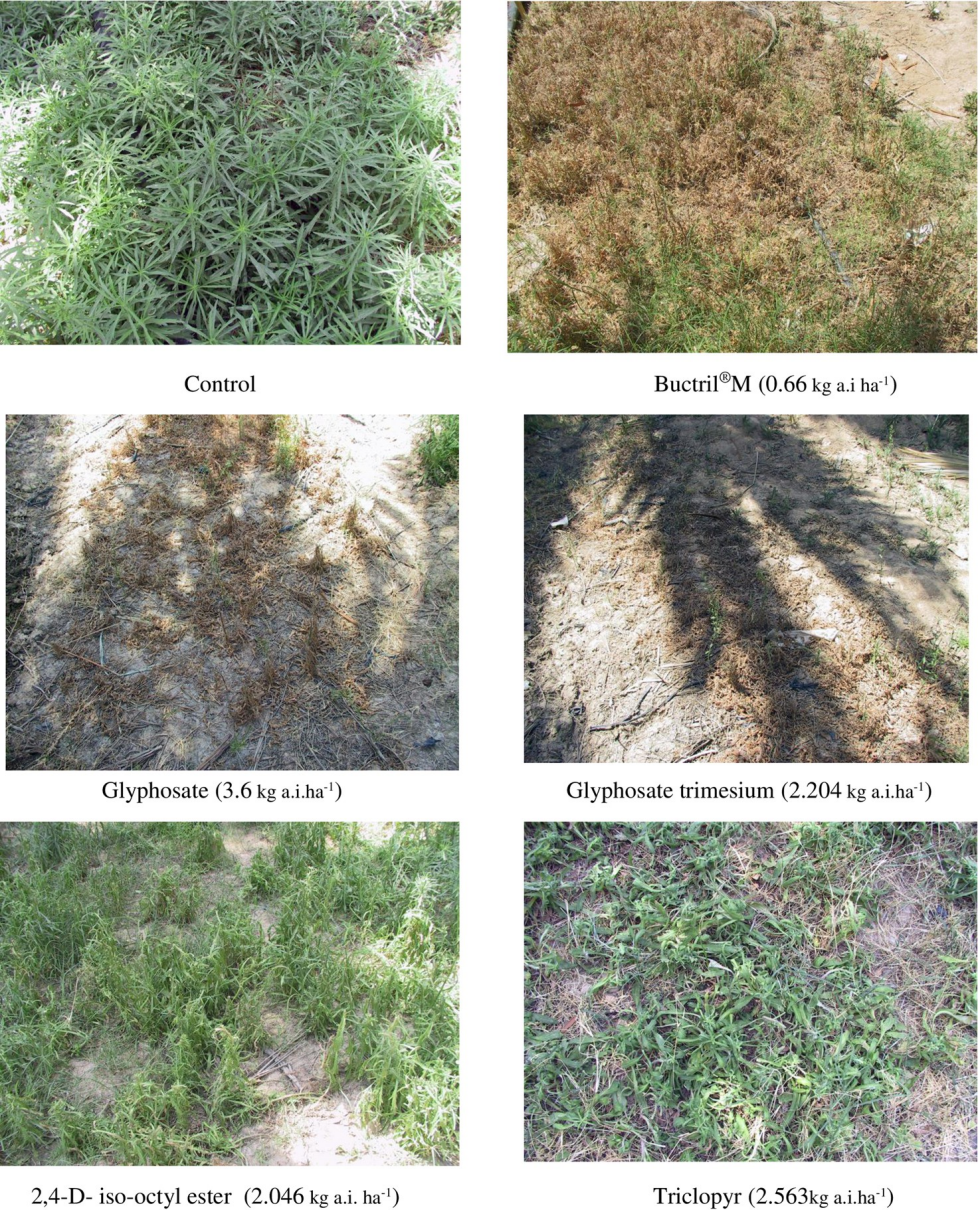
Most effective herbicides used at normal recommended rates of applications against *Erigeron bonariensis* at 15 DAT.

**Fig 3:**
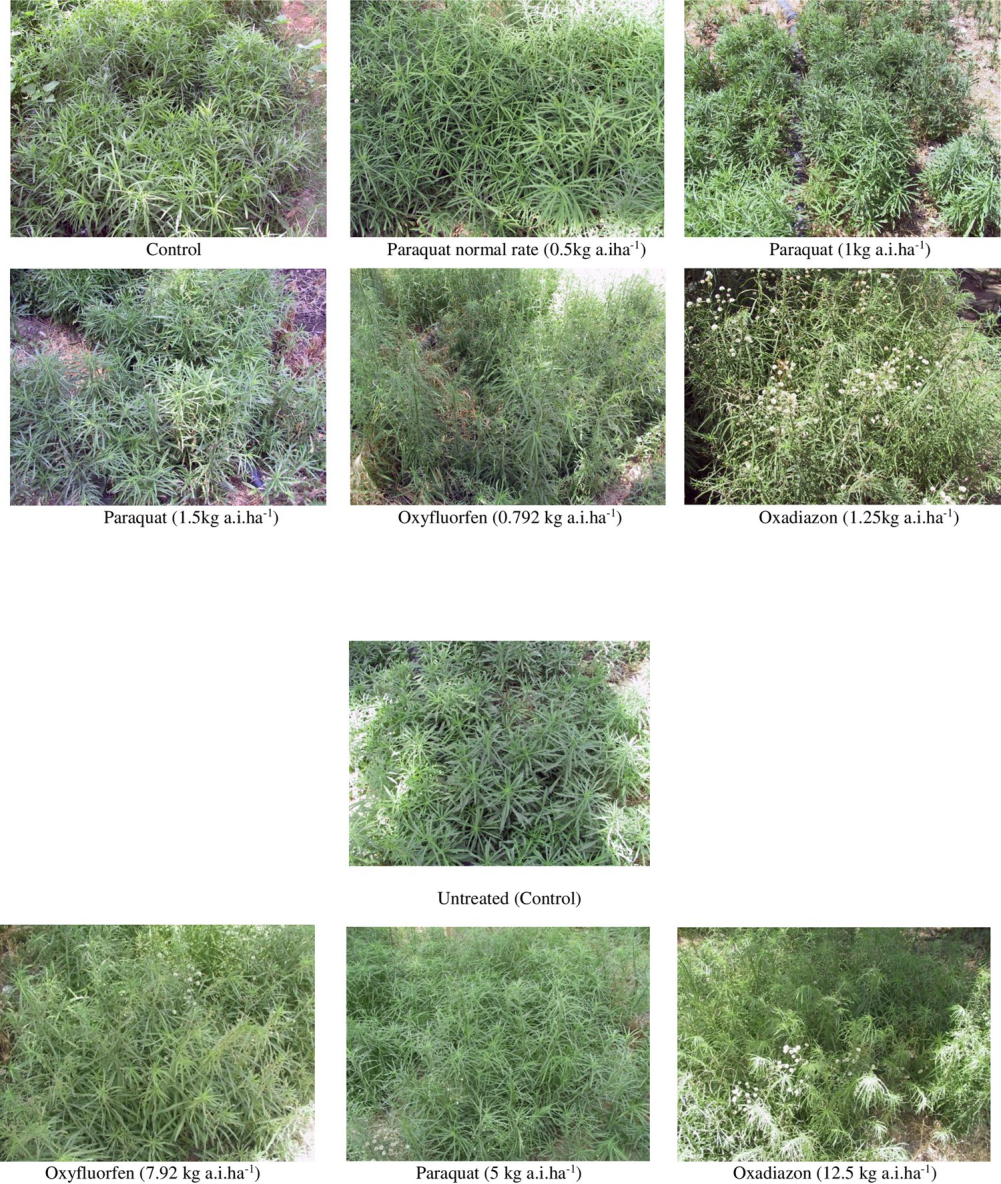
Effect of oxyfluorfen, paraquat and oxadiazon used at normal and 10- fold of the recommended rates of application on *Erigeron bonariensis* at 15 DAT.

Visual estimation of the effects of all herbicides treatments carried out at three weeks after application showed bromoxynil/MCPA mixture (Buctril^®^M), triclopyr and glyphosate had the highest phytotoxic effects (0 score for each) and completely killed weed plants (Table 3 and Fig 2) and these followed by 2,4-D- iso-octyl ester (0.3), glyphosate trimesium (1.0) and ioxynil octanoate (1.0). The effect of mecoprop was much improved (2.8) at this evaluation date but bentazon effect was slightly improved (3.0). However, the effect of paraquat used at increasing rates of application (0.5, 1 and 1.5 kg a.i. ha^-1^), oxadiazon (1.250 kg a.i.ha^-1^) and oxyfluorfen (0.792kg a.i.ha^-1^) remained the same (10, 9.5 and 10, respectively) with no obvious phytotoxic effect on weed (Table 3 and Fig 3). Growth of *E*. *bonariensis* plants showed some differences between the two evaluation dates at which plants treated with Mecoprop, ioxynil octanoate and Linuron+terbutryn (Tempo) showed more death at the second evaluation date while plants treated with oxadiazon showed some recovery from herbicide phytotoxicity (Table 3).

**Experiment 2.** Testing the highest rates (5, 12.5 and 7.92 kg a.i. ha^-1^) of paraquat, oxadiazon and oxyfluorfen herbicides, respectively failed to control *E*. *bonariensis* (Table 4). At two weeks after application, none of the rates used for any of the three herbicides affected *E*. *bonariensis* growth (8, 9 and 9, respectively) but caused somehow a slight change in green color of the plants with some burning on leaf tips in oxadiazon and oxyfluorfen treated plots (Fig 3). However, weed plants appeared normal (9, 10 and 10, respectively) and restored their green color at three weeks after application (Table 4 and Fig 3).

**Table 4:** Visual estimations of the effects of selected foliage applied herbicides on *Erigeron bonariensis* in date palm field, values are for two estimations conducted at 15 and 23 days after herbicides treatments (DAT).

Herbicides	Rate of application (a.i.kgha^-1^)	Score at 15 DAT	Score at 23 DAT
Untreated (Control)	0	10.0^a^	10.0^a^
Paraquat	5.00	8.0^b^	9^b^
Oxyfluorfen	7.92	9.0^ab^	10^a^
Oxadiazon	12.50	9.0^ab^	10^a^
LSD ≤ *p* (0.05)	-	1.3	0.7

Mean values for three replicates treated with each herbicide on a scale of 0–10 where 0 denotes that the weed was completely controlled and the weed had no possible recovery after treatment, and 10 means that the weed was in full growth and no herbicide phytotoxicity observed. Mean values in the same column followed by the same lower-case letter are not significantly different according to Fisher’s LSD at *P* = 0.05.

## Discussion

Hairy fleabane (*E*. *bonariensis*) populations in certain parts of the Jordan Valley are rapidly expanding in the last few years. Annual weeds are usually controlled by hand weeding, tillage, soil mulch and the use of general contact herbicides. However, in addition to paraquat that is widely used in orchards, other herbicides of different modes of action were tested against paraquat-resistant *E*. *bonariensis*. Glyphosate resistance had been reported previously for this weed in various regions globally [13, 15, 16, 18, 26].

A preliminary glasshouse experiment on two populations of *E*. *bonariensis* collected from two different sites in the Jordan Valley revealed that the date palm weed population was highly resistant to paraquat, oxadizone and oxyfluorfen herbicides used at normal and 10-fold higher rate of application and when herbicides application was repeated. The other weed population (at al-Twal site) was highly susceptible to the three herbicides and at all rates used.

Our results in the field show that paraquat at normal and higher rates of application in the first field experiment failed to harm the weed. Our findings are similar to cases of paraquat resistance reported elsewhere [17, 18, 27]. Other contact herbicides oxadiazon and oxyfluorfen are commonly used by local farmers for weed control in vegetables (mainly in onions) as well as in fruit trees and for general weed control in uncultivated lands in different parts of the country. This may have led to a buildup of resistance in common weed species or populations to these herbicides. The *Erigeron bonariensis* population in the date palm orchard we tested were highly resistant to the three herbicides although some other less used herbicides such as Linuron + terbutryn formulation (Tempo*) showed slight harmful effects on the weed. The ineffectiveness of oxyfluorfen contradicts the results of Travlos & Chachalis [28] who reported that this herbicide is useful for managing resistant *E*. *bonariensis* in a mixture with glufosinate suggesting the effect was attributable to the glufosinate.

In contrast, bromoxynil/MCPA mixture (Buctril^®^M), was completely controlled *E*. *bonariensis* plants at 5 days after application and treated plants failed to recover. This mixture is made from bromoxynil (which has a contact action) mixed with MCPA (which is a systemic herbicide) and both are formulated in a mixture commercialized as Buctril^®^M. MCPA has been reported as an excellent herbicide to control the same weed species at all leaf stages and all weed biotypes could be effectively controlled [29]. Glufosinate is a contact herbicide with some systemic action and has been shown to be effective against glyphosate and paraquat-resistant populations of *E*. *bonariensis* [18]. In addition, diquat, glufosinate, or glufosinate + oxyfluorfen controlled glyphosate-resistant or -susceptible *E*. *bonariensis* [28]. These herbicides, along with various integrated management strategies, have good potential to manage or slow the spread of glyphosate resistance in this weed species.

Our results demonstrated that glyphosate and glyphosate trimesium (Trimethylsulfonium *N*-[(hydroxyphosphinato) methyl] glycine) were highly effective and almost completely controlled the weed at 2^nd^ evaluation date. However, both showed complete weed control a week later, likely because of slower systemic action of these herbicides. *Erigeron bonariensis* has evolved resistance many times [11–13, 15, 16, 18, 26]. The effectiveness of these two forms of glyphosate suggests that the chemical was not widely used prior to the ban of paraquat use in the country. Glyphosate was only used to control perennials that are difficult to control by other herbicides or means. Therefore resistance to this herbicide may not have developed yet in Jordan. 2,4-D and triclopyr were both effective and almost completely controlled plants at the normal recommended rates. However, these took longer than other herbicides to fully exert their harmful effects on the weed which is normally expected for translocated herbicides. These chemicals however, had their own specific mode of action observed at first evaluation time which included leaf malformation, twisting, growth abnormality and curling. Some of these chemicals however, have been recommended by other workers [30] for use against the same weed species either alone or in mixture with glyphosate or picloram. Mixtures of glyphosate with saflufenacil and glyphosate with 2,4-D were reported as effective in controlling susceptible, glyphosate-resistant, and glyphosate-paraquat-resistant populations of *E*. *bonariensis* [18]. Glyphosate-resistant weeds were effectively controlled using 2,4-D and dicamba [31] and the weed responded differently to both herbicides. It is worth mentioning that both MCPA and 2,4-D and trichlopyr are of related groups and no kind of resistance to any of these herbicides have been developed or reported in this weed species [5].

Further testing of paraquat, oxadiazon and oxyfluorfen showed that resistance to these chemicals by *E*. *bonariensis* is well developed and very high application rates of the three herbicides had no appreciable harmful effects on the weed. All treated plants maintained normal growth similar to those of untreated control. Resistance of the weed to oxyfluorfen and oxadiazon herbicides represents the first report on *E*. *bonariensis* resistance to these chemicals. The use of other herbicides of different mode of action such as desiccants (bromoxynil/MCPA mixture) or systemic cause malformation, twisting and growth abnormalities (2,4-D and trichlopyr) or burning systemic (glyphosate formulations) is necessary to mitigate the spread of resistant population and glyphosate and phenoxy herbicides have an important role at this stage either used separately, in combination or when integrated with other methods of weed control.

## Conclusions

Adoption of integrated management of *Erigeron* is essential to ensure that plants of this fast adapting genus will remain under control in arable and perennial crops. Farmers certainly are unfamiliar with *E*. *bonariensis* resistance to paraquat, oxadiazon and oxyfluorfen herbicides that commonly applied in different parts of the country. However, this is the first report on resistance of *E*. *bonariensis* and the second case of weed resistance in Jordan after *Convolvulus arvensis* being the first perennial weed resistant to paraquat in dates. Since paraquat, oxadiazon and oxyfluorfen in this study are widely used in the country and most likely included in weed control programs, therefore steps should be taken to prevent the development of resistant populations of this common weed species, species of the same genus and of other weed species. Agricultural practices should aim at preventing the spread of herbicide-resistant populations and reducing the buildup of resistance to the widely used herbicides. This however, can only be achieved through herbicide rotation to include the effective herbicides (bromoxyni/MCPA mixture, glyphosate and trichlopyr) against the weed reported in this study, using other weed control methods such as mechanical and cultural or at least minimizing the use of these resisted herbicides by the weed in places where *E*. *bonariensis* is prevailing and commonly spread. Label resistance warning should be also indicated on all products. All herbicidal control options used against *E*. *bonariensis* however, should be integrated with other weed control methods in order to prevent escalating populations’ resistance.
